# Effectiveness of peer-assisted teaching of medical English skills to non-native English-speaking medical students

**DOI:** 10.12688/mep.19694.2

**Published:** 2023-12-28

**Authors:** Ahmad Al Shihabi, Heba Mardini, Ahmad N. Alkhaledi, Lana Jarad, Rama Jaber, Ramez Jaber, Sara Naoura, Mohammad Bashar Izzat

**Affiliations:** 1 Department of Surgery, Damascus University Faculty of Medicine, Damascus, Damascus, Syria

**Keywords:** medical education, peer teaching, medical students, English language

## Abstract

**Background:**

Peer-assisted learning has been shown to be constructive in numerous aspects of undergraduate medical education. The purpose of this study was to evaluate the effectiveness of peer-assisted teaching of medical English skills to non-native English-speaking students.

**Methods:**

A medical English conversation course was conducted at Damascus University by a group of students. Targeted participants were intermediate level fellow students from the same program. A longitudinal study was carried out between 1 ^st^ to 31 ^st^ March 2019 to assess changes in self-assessment of English language skills among course participants. Pre- and post-course appraisal involved a review of previous experience with medical English language, a self-assessment of five English language skills, and an objective measurement of medical English knowledge. In addition, participants were requested to respond to a set of statements related to the importance and the usefulness of peer-assisted teaching of medical English skills. Paired-sample Student *t*-test was used to compare pre- and post-course appraisal results.

**Results:**

42 students attended the course and completed pre- and post-course appraisals in full. Data analyses showed a statistically significant increase in participants’ confidence in speaking medical English in public ( *p*<0.001) and using English in various medical settings (presenting and discussing cases, writing clinical reports, interviewing patients and reading English medical texts). Objective measurements of medical English knowledge confirmed a significant increase in participants’ knowledge of methods of administration of therapeutics, knowledge of human body parts in English and familiarity with English medical abbreviations. Most participants agreed that peer-education was effective in teaching medical English skills to non-native English-speaking students and in increasing their confidence when using English in real-life medical scenarios.

**Conclusions:**

The present study highlights the effectiveness of peer-assisted teaching of medical English skills to non-native English-speaking medical students. Further validation is required and should compare the effectiveness of traditional versus peer-assisted teaching approaches.

## Introduction

English continues to be the language of choice for medical teaching, conferences, and publishing. Graduates of overseas medical schools that teach medicine in native languages and wish to practice medicine in English-speaking countries are confronted with the multi-faceted challenge of having to interact with local patients using customary colloquial jargons, and to interconnect with peers with accurate medical vocabulary as well ^1– 3^. Moreover, a multitude of English linguistic skills ought to be acquired in order to be able to obtain up-to-date knowledge, perform state of the art treatments and communicate with an increasing number of international colleagues, the most demanding of which may be writing and presentation skills which are also necessary for success and progression in academic medicine ^4– 7^. Several strategies have been proposed to help non-native English-speakers improve their English language skills, such as engaging in verbal exercises, one-to-one sessions with faculty members, academic writing support workshops and the development of interactive computer-simulation materials ^8– 11^.

Peer-assisted learning (also known as ‘peer-education’) is the method of exchanging knowledge between people who are at a similar stage in their academic education ^12^. Teachers and students in this setting share similar knowledge base, learning experiences, and social roles. This helps selecting the most appropriate teaching means and promotes student leadership ^13, 14^ and has been shown to be constructive in numerous aspects of undergraduate medical education ^15^.

The aim of this project was to evaluate the effectiveness of peer-assisted teaching of medical English skills to non-native English-speaking students from the Clinical Sciences program at the Faculty of Medicine, Damascus University. An earlier version of this study can be found on ResearchSquare ^16^.

## Methods

### Ethical statement

The study protocol was approved by the Research and Ethics Committee at the Faculty of Medicine, Damascus University (ref 3/Med/2019). Before course commencement, informed consent to participate and for publishing this study were signed by all course participants who were all above the age of 16 years. Participants’ data was protected by removing all identifying information before data is transferred and reviewed, and by implementing robust data security standards. The Medical English Conversation course was also approved by the undergraduate dean prior to implementation.

### Study design

This was a longitudinal study using a within-subjects design to assess changes in self-assessment of English language skills among course participants.

### Study setting

This research project was carried out at the Faculty of Medicine, Damascus University, which runs a six-years’ undergraduate program in Medicine. This program is divided into three years tutoring in basic sciences (biology, chemistry, physics, psychology, and sociology) followed by three years training in clinical sciences, upon completion of which a Doctor of Medicine degree is awarded. 

A group of final year medical students from the Clinical Sciences program designed and conducted a dedicated Medical English Conversation course. This was based upon prior experience with peer education courses at the Faculty of Medicine, with input from an experienced English-speaking faculty member (MBI). Targeted participants were fellow fifth and sixth year medical students from the Clinical Sciences program. 12 topics were selected to make up the course curriculum, which are:

1- Medical literature

2- History taking

3- Case presentation

4- Parts of the body

5- Communication skills

6- Ethics

7- Investigations

8- Medicaments

9- Hospital departments and medical records

10- Reading skills

11- Abstracts and research articles

12- Presentation skills

These topics were organized into eight sessions, each lasting 90 minutes, to be delivered over four consecutive weeks.

### Course admission and conduct

**Figure 1.**
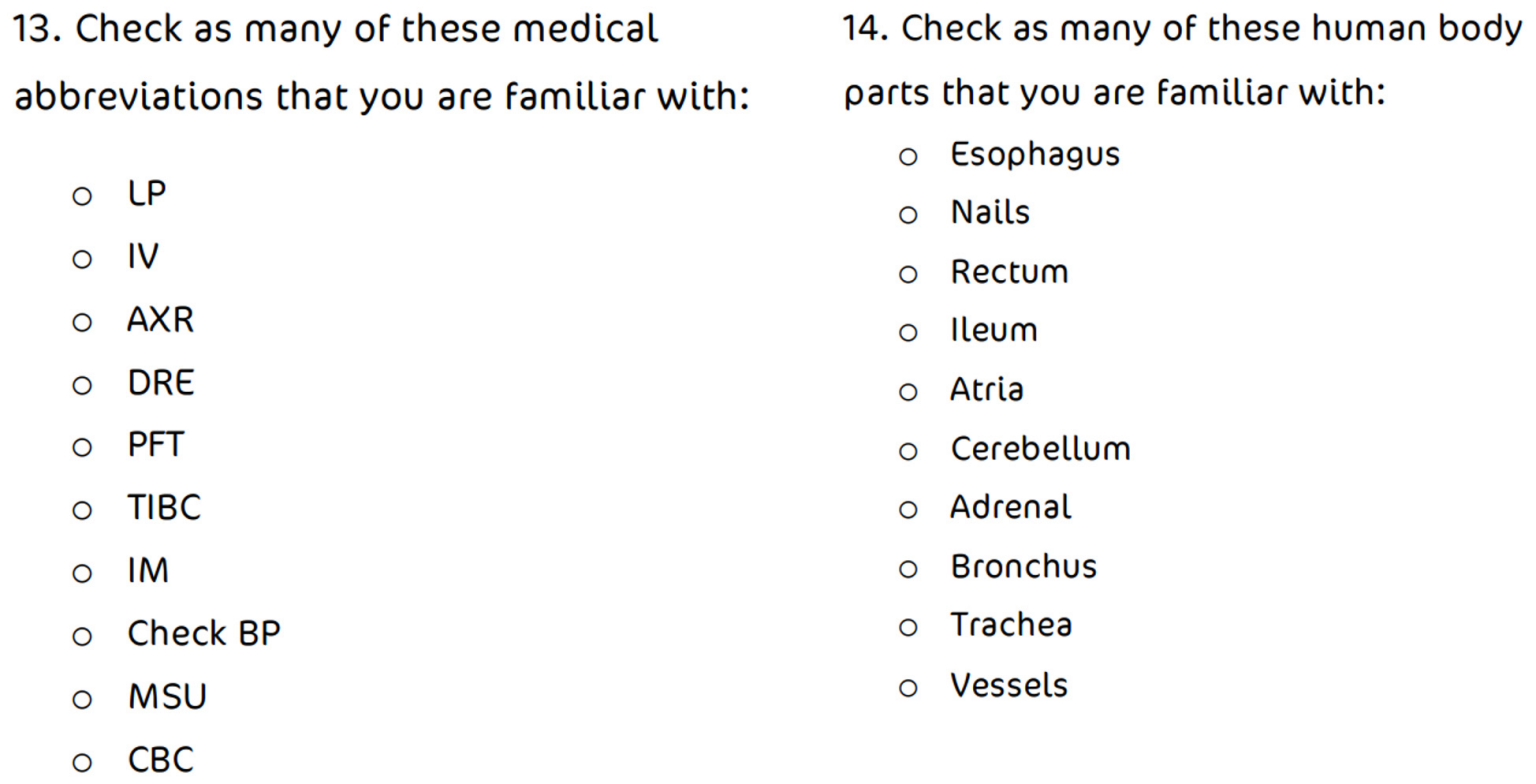
Participants were asked to check medical abbreviations and parts of the body which they are familiar with.

**Figure 2.**
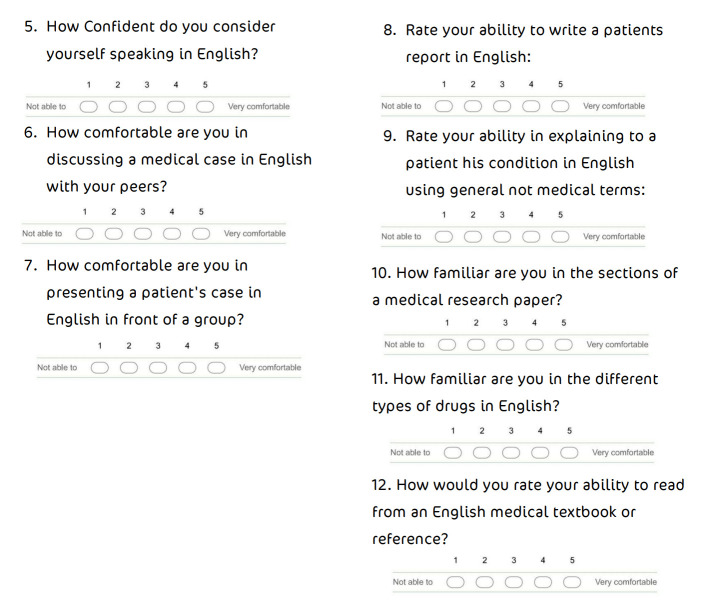
Self-assessment of comfort and confidence in several areas.

The Medical English Conversation course was publicized on social media, and interested applicants were required to complete a three-elements pre-admission appraisal. The first element was a self-assessment of five English language skills (case presentation, writing a clinical report, explaining details to patients, familiarity with sections of a research paper, and reading an English medical text). The second element was a review of previous experience with medical English language (attending medical English courses, public presentations in English, electives in an English-speaking country, clinical rounds or history-taking, writing research projects or reading English articles). For this element, the five-point Likert scale was used to measure attitudes directly, with one referring to ‘very uncomfortable’ and five referring to ‘very comfortable’. The third element was an objective measurement of medical English knowledge (knowledge of methods of administration of therapeutics and human body parts in English) along with a self-report of familiarity with a list of English medical abbreviations. For this element, applicants were asked to indicate terms that they are familiar with from given vocabulary [ Table 1, Figure 1– Figure 2]. This questionnaire was validated beforehand through distributing it among a group of medical students who did not join the course, but whose English language is very good according to external placement tests at local English language institutes. The questionnaire was then reviewed by facilitators who checked the conformity of the questions with set targets.

**Table 1.** The three-elements’ pre-admission appraisal.

Element	Contents
1	Demographics and self-assessment of English language skills
	• Overall assessment (excellent, good, intermediate, weak) • case presentation • writing a clinical report • explaining to patients • familiarity with sections of a research paper • reading an English medical text • Previous general English language courses
2	Previous experience with medical English language
	• Previous medical English courses • Previous public presentations in English • Electives in an English-speaking country • Clinical rounds or history-taking • Writing research projects • Reading English textbooks and articles
3	Objective measurement of medical English knowledge
	• Knowledge of methods of administration of therapeutics • Knowledge of human body parts in English • Familiarity with a list of English medical abbreviations ( Figure 1)
	Self-report of familiarity with a list of English medical abbreviations.

The selection of course participants was based on this appraisal and admission was focused on ‘intermediate level’ applicants according to external placement tests at local English language institutes which had to be taken no more than one year before joining the course. There were no cutoff scores of students in our questionnaire or cutoff scores in other standardized English evaluation tests such as TOEFL or IELTS, but we rather did a holistic review of the applicants in order to select candidates with sufficient basic English skills to enroll in the course. Accepted applicants were then requested to respond to a set of statements related to the importance of medical English skills for doctors from non-English-speaking countries. The five-point Likert scale was used to measure attitudes directly, with one referring to ‘strongly disagree’ and five referring to ‘strongly agree’.

Participants were organized into groups of four to five students, and each group was assigned a final year medical student from the Clinical Sciences program who was deemed to have appropriate English language experience to qualify as a ‘peer-teacher’. This required having attended a four-weeks’ clinical elective at the American University of Beirut Medical Center (conducted in English), in addition to achieving at least an upper-intermediate level in the standardized English tests of the Damascus University Languages Center. In addition, every peer teacher was enrolled in a one-day-training on facilitation, motivation and general considerations in teaching English language.

The course took place between the beginning of April and end of May 2019. All sessions were held in university lecture rooms except for one ‘field session’ which was held at the university hospital to allow practical application of given concepts. Each session lasted 90 minutes and incorporated interactive teaching methods (workshops, group discussions, role-playing scenarios, etc.). In addition, daily medical topic discussions were held on social media (a Facebook group) among all course participants. Each participant was required to write one communication every week relating to a chosen topic, and other participants were asked to discuss those topics and comment in writing.

**Figure 3.**
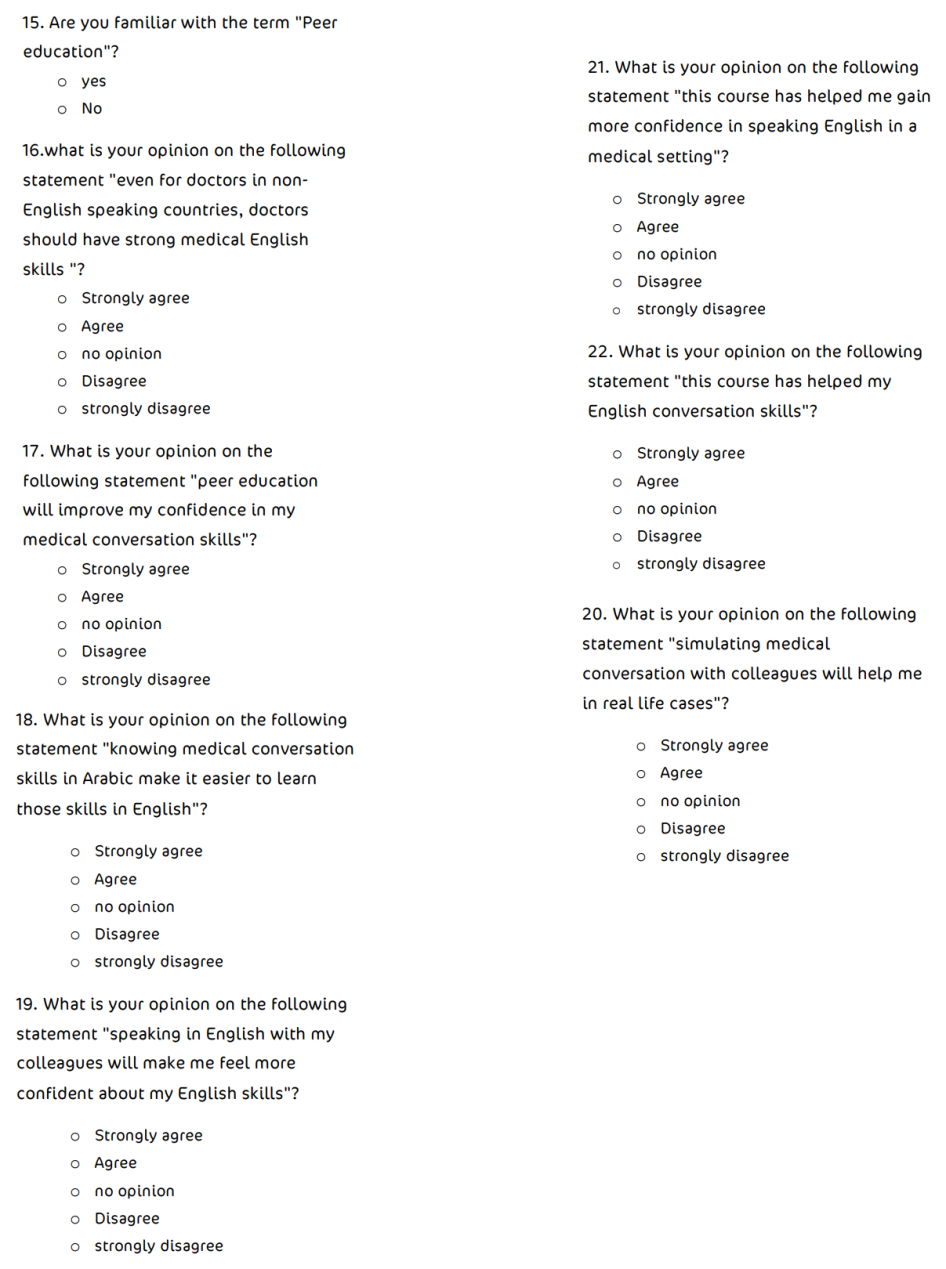
Participant opinion regarding peer-assisted learning.

Upon completion of the course, participants were asked to re-fill the three-elements’ appraisal and to respond again to the previous set of statements. In addition, participants were requested to respond to a set of statements related to the usefulness of peer-assisted teaching of medical English skills in real life scenarios [ Figure 3]. The five-point Likert scale was used again to measure attitudes directly, with one referring to ‘strongly disagree’ and five referring to ‘strongly agree’.

### Statistical methods

Data was entered in Microsoft Excel 2020 and exported to IBM SPSS v.26 ( https://www.ibm.com/spss) for both descriptive and inferential statistical analysis. PSPP software application ( http://www.gnu.org/software/pspp/) is also able to run the same analysis in this study. Paired-sample Student *t*-test was used to compare pre-admission and post-course self-assessments of English language skills. Independent sample Student *t*-test and ANOVA tests were used to correlate responses with students’ demographics. Statistical significance was indicated by *p*<0.05.

## Results

84 students from the Clinical Sciences program applied to the Medical English Conversation course and completed the pre-admission questionnaire, of whom 48 were accepted to the course and attended all sessions. 42 students (87.5%) completed both pre-admission and post-course appraisals in full and they comprised our study group, while four students did not finish the course due to transportation issues and two due to personal reasons. Demographics and pre-admission appraisals are shown in Table 2. Most participants (85%) projected their English language skills to be intermediate or good. Even though 45% of them had previously attended general English language courses, experience with medical English was largely limited to reading articles written in English.

**Table 2.** Results of demographics and pre-admission appraisal.

Element	Contents	n (%)
1	Gender (male/female)	15/27 (36/64 %)
	Current year in the Clinical Sciences program	
	1 ^st^ year	23 (55%)
	2 ^nd^ year	11 (26%)
	3 ^rd^ year	8 (19%)
	Self-assessment of English language	
	excellent	2 (5%)
	Good	21 (50%)
	Intermediate	15 (35%)
	weak	4 (10%)
	Previous general English language courses	19 (45%)
2	Previous experience with medical English	
	Previous medical English courses	5 (12%)
	Previous public presentations in English	12 (29%)
	Electives in an English-speaking country	2 (5%)
	Clinical rounds or history-taking	7 (17%)
	Writing research projects	11 (26%)
	Reading English books and articles	37 (88%)

Answers to the five-point Likert scale before and after the course were compared using the paired-sample *t*-test. This showed a statistically significant increase in participants’ confidence in speaking medical English in public, with an increase in the percentage of students responding ‘comfortable’ or ‘very comfortable’ from 26% prior to attending the course to 50% afterwards ( *p*<0.001). Similarly, self-assessments of participants’ confidence in using English in other medical settings (presenting and discussing cases, writing clinical reports, interviewing patients, and reading English medical texts) showed a statistically significant increase in students’ confidence levels [ Table 3].

**Table 3.** Percentage of confident responses to the five-point Likert scale before and after the course.

English language skills	Pre-	Post-	P *
Presenting a case	4 (9%)	25 (59%)	<0.001
Writing a clinical report	5 (12%)	21 (50%)	<0.001
Explaining to patients	8 (19%)	24 (57%)	<0.001
Familiarity with sections of a research paper	12 (29%)	21 (50%)	<0.001
Reading an English medical text	27 (64%)	35 (84%)	<0.05

* paired-sample t-test

Given the unavailability of standardized and objective tests of medical English language skills (such as the OET medicine) in our country, we attempted to use a set of questions in our pre- and post-course questionnaire that are far from the self-assessment of competence, such as identifying correct medical terminologies. This fairly objective measurement of medical English knowledge confirmed a significant increase in participants’ knowledge of methods of administration of therapeutics and of human body parts in English. In addition, participants reported better familiarity with a list of English medical abbreviations [ Table 4].

**Table 4.** Objective measurements and self-reports of English knowledge before and after the course.

English language skills	Pre-	Post-	P *
Knowledge of methods of administration of therapeutics	13 (31%)	17 (41%)	<0.05
Knowledge of human body parts in English	26 (62%)	41 (98%)	<0.001
Self-report of familiarity with ˃50% of given English medical abbreviations	18 (43%)	33 (79%)	<0.001

* paired-sample t-test

Changes in responses to the set of statements among course participants are shown in Table 5. Students unanimously agreed that medical English skills are necessary for doctors in non-English speaking countries. Importantly, most course participants (88%) agreed that peer-education was effective in teaching medical English skills to non-native English-speaking students and in increasing their confidence when using English in real-life medical scenarios [ Table 6]. No significant correlation was found between students’ demographics or previous experiences and any of the responses.

**Table 5.** Statements related to the importance of medical English skills for doctors from non-English-speaking countries.

Statement		agree	no opinion	disagree	p
1. Doctors in non-English speaking countries need adequate medical English skills	Pre-	90%	5%	5%	p<0.05
	Post-	100%	0%	0%	
2. Being skillful in medical discussions in my native language makes it easier to acquire those skills in English	Pre-	69%	17%	14%	ns
	Post-	69%	10%	21%	
3. Talking in English to my colleagues boosts my confidence in my English skills	Pre-	88%	2%	10%	ns
	Post-	88%	0%	12%	
4. Peer education will boost my confidence in my medical English skills	Pre-	79%	12%	10%	ns
	Post-	83%	5%	12%	

**Table 6.** Responses to statements related to the usefulness of peer-assisted teaching.

Statement	agree	no opinion	disagree
1. Simulating medical discussions with colleagues will help me in real life scenarios	81%	7%	12%
2. This course helped me gain more confidence in speaking English in a medical setting	88%	2%	10%
3. This course helped me improve my English conversation skills	76%	10%	14%

## Discussion

The demand for medical English education in countries where medical school instruction is carried out purely in native languages has been rising with globalization ^10^. Conducting medical education purely in native languages is believed to lessen graduates’ ability to absorb and to contribute to scientific advances, and to hold back opportunities for international collaboration among physicians and researchers ^17^. On the contrary, a sound medical English curriculum can support students’ lifelong independent pursuit of medical knowledge. Consequently, proficiency in medical English grew to be a crucial pre-requisite for medical graduates ^18^.

Medical English education in our country has traditionally been conducted through didactic lectures, focusing on basic medical terminology as well as on reading and writing skills. This method, however, is ineffective in promoting English communication and may decrease students’ motivation ^10^. As a result, recent guidelines concerning medical English curriculum development called for a shift from repetition and mastering grammar toward an emphasis on functional, communication-oriented teaching and the development of listening and speaking skills ^19^.

Peer-assisted methodologies have been utilized successfully for teaching certain medical skills, such as basic life support and basic surgical skills ^20, 21^, and it has been shown that medical students perceive peer-teaching to be appropriate and beneficial when implemented in medical education ^22^. For example, a previous study showed that students preferred to discuss conceptual problems with peers who were closer to them than with faculty teachers who may not understand their reasons for difficulty with a subject matter, or who might have limited interest in communicating with novices ^23^. Peer teachers were able to create a comfortable and safe educational environment, provide access to role models, and enhance intrinsic motivation to study ^23^.

Literature pertaining to peer-assisted teaching of medical English skills remains very limited ^24^. The present preliminary study validates the effectiveness of peer-assisted teaching of medical English skills to non-native English-speaking medical students. The Medical English Conversation course focused on motivating students to learn English by sharing experiences, creating opportunities and helping them envisage themselves in a clinical environment. The course attempted to familiarize participants with medical English terminologies in line with words used in colloquial jargon as this would support effective communication with both patients and colleagues. Peer education was appropriate for this purpose. Participants not only understood the terminologies but were also able to explain them to their peers during their presentations using simple terms. This is likely to reinforce better understanding and retention of knowledge ^24^. Similar to previous reports ^24^, our study shows that participants’ knowledge of English medical terminologies and abbreviations increased significantly, with a substantial improvement in participants’ confidence in using English in various real-life medical settings, including presenting and discussing cases, writing clinical reports, interviewing patients, and reading English medical texts.

### Study limitations

One limitation to this study is the lack of objective evaluations of medical English skills, and the use of subjective changes in self-assessments among course participants to validate the effectiveness of peer-assisted teaching. An inherent bias may exist in this methodology as enthusiastic participants are likely to report an increase in their confidence in using English immediately following the course. This does not take into account the decay of information over time where early enhancements do not translate into actual improvements in English language skills later on or when put into real hospital environment.

A second limitation is that it was difficult to analyze or draw attention to a key outcome of all medical programs, which is patient satisfaction, but this was not possible as the patient population is non-English speaking. Hence, our assessment was carried out using peer-to-peer simulation rather than getting real-time feedback from actual patients.

Finally, this study was conducted in a country where medical students are prominently engaged in foreign language learning activities in order to advance their immigration potential. Further studies are needed to establish the effectiveness of peer education and its application in other societies where medical students may not be so enthusiastically engaged in English language learning activities.

## Conclusions

The medical profession is becoming even more competitive, embracing scholar-centeredness and continued learning pursuit. The present preliminary study verifies the effectiveness of peer-assisted teaching of medical English skills to non-native English-speaking medical students. Further validation is required and should implement objective evaluations of medical English skills, both within-subjects and between-subjects, and should compare the effectiveness of traditional versus peer-assisted teaching approaches.

## Data Availability

Figshare: Effectiveness of peer-assisted teaching of medical English skills to nonnative English-speaking medical students.xlsx. https://doi.org/10.6084/m9.figshare.23537397.v1 ^25^. This project contains the following underlying data: Effectiveness of peer-assisted teaching of medical English skills to nonnative English-speaking medical students.xlsx. (Anonymised pre-and post- test results). Data are available under the terms of the Creative Commons Attribution 4.0 International license (CC-BY 4.0). Figshare: MEC Registration form. https://doi.org/10.6084/m9.figshare.23619045.v1 ^26^. This project contains the following extended data: MEC registration form.pdf. (Blank copy of MEC form). Data are available under the terms of the Creative Commons Attribution 4.0 International license (CC-BY 4.0).
