# Supplementary material for: Effectiveness of peer-assisted teaching of medical English skills to non-native English-speaking medical students
Source: MedEdPublish (2016). 2023 Dec 28;13:36. Originally published 2023 Jul 19. [Version 2] doi: 10.12688/mep.19694.2 (PMC10831228; doi:10.12688/mep.19694.2)
Supplement: Peer review history

# Reviewer report: Reviewer response for version 2

**DOI:** 10.21956/mep.21529.r35715

**Published:** 2024-01-31

**Authors:** Theodora Tseligka

**Affiliations:** 1 University of Ioannina, Ioannina, Greece

The authors have addressed most of the points raised in the previous version.  I still do not find the description "objective measurements of medical English knowledge" an acceptable one, since there does not seem to be an 'objective' way in the selection of these abbreviations and terms. They appear to be randomly selected. 

 Overall, though, the authors have put a considerable effort in this study.

Have any limitations of the research been acknowledged?

Partly

Is the work clearly and accurately presented and does it cite the current literature?

Partly

If applicable, is the statistical analysis and its interpretation appropriate?

Partly

Are all the source data underlying the results available to ensure full reproducibility?

Partly

Is the study design appropriate and is the work technically sound?

Partly

Are the conclusions drawn adequately supported by the results?

No

Are sufficient details of methods and analysis provided to allow replication by others?

Partly

Reviewer Expertise:

Medical English, Narrative Medicine, FL policies in Higher Education, Digital Discourse

I confirm that I have read this submission and believe that I have an appropriate level of expertise to confirm that it is of an acceptable scientific standard.

# Reviewer report: Reviewer response for version 2

**DOI:** 10.21956/mep.21529.r35714

**Published:** 2024-01-08

**Authors:** Anne D Souza

**Affiliations:** 1 Department of Anatomy, Kasturba Medical College, Manipal, Manipal Academy of Higher Education, Manipal, Karnataka, India

The authors have addressed all the points with clear explanations. I do not have any further comments.

Have any limitations of the research been acknowledged?

Yes

Is the work clearly and accurately presented and does it cite the current literature?

Yes

If applicable, is the statistical analysis and its interpretation appropriate?

Yes

Are all the source data underlying the results available to ensure full reproducibility?

Yes

Is the study design appropriate and is the work technically sound?

Yes

Are the conclusions drawn adequately supported by the results?

Yes

Are sufficient details of methods and analysis provided to allow replication by others?

No

Reviewer Expertise:

Simulation, 3D printing in medical education, ethics in medical education, designing and planning learning

I confirm that I have read this submission and believe that I have an appropriate level of expertise to confirm that it is of an acceptable scientific standard.

# Reviewer report: Reviewer response for version 1

**DOI:** 10.21956/mep.21095.r34719

**Published:** 2023-09-07

**Authors:** Theodora Tseligka

**Affiliations:** 1 University of Ioannina, Ioannina, Greece

The present paper aims to report on the effectiveness of a valuable initiative, namely peer-assisted teaching to promote Medical English skills among non-native Medical English students. Despite the clear structure and organisation of the paper, there are major drawbacks that need to be considered before finally accepting the submission of this paper.  

- A 1-month study does not really qualify as **a longitudinal study.**
- [Introduction]: “are **faced** with the **multi-faceted** challenge” – repetition
- More updated references are required – and some key ones in the field are missing
- Course admission and contact: “The selection of course participants was based on this appraisal and admission was focused on ‘intermediate level’ applicants according to external placement tests at local English language institutes which had to be taken no more than one year before joining the course.” The selection process is not clear. What exactly do you mean that selection was based on this self-appraisal? Who were eventually selected? Did they need to score a specific result in this scale? And then, how does this correlate with their scores in the English language tests prior to the study?
- What exactly were these “Objective measurements of medical English knowledge”? Please describe the exact tool. And then, in the limitations, why do you mention that there were NO objectives measurements? Please clarify.
- “Medical English education has traditionally been conducted through didactic lectures, focusing on basic medical terminology as well as on reading and writing skills.” This is kind of outdated. A lot has progressed in Medical English teaching in recent decades, with the integration of web technologies and innovative methodologies.
- “A second limitation is that it was difficult to draw attention to hard outcomes, such as patient satisfaction, as the patient population is non-English speaking, which makes the validation of the tool hard to achieve.” Unclear. Please elaborate.

Have any limitations of the research been acknowledged?

Partly

Is the work clearly and accurately presented and does it cite the current literature?

Partly

If applicable, is the statistical analysis and its interpretation appropriate?

Partly

Are all the source data underlying the results available to ensure full reproducibility?

Partly

Is the study design appropriate and is the work technically sound?

Partly

Are the conclusions drawn adequately supported by the results?

No

Are sufficient details of methods and analysis provided to allow replication by others?

Partly

Reviewer Expertise:

Medical English, Narrative Medicine, FL policies in Higher Education, Digital Discourse

I confirm that I have read this submission and believe that I have an appropriate level of expertise to confirm that it is of an acceptable scientific standard, however I have significant reservations, as outlined above.

# Reviewer report: Reviewer response for version 1

**DOI:** 10.21956/mep.21095.r34257

**Published:** 2023-09-07

**Authors:** Anne D Souza

**Affiliations:** 1 Department of Anatomy, Kasturba Medical College, Manipal, Manipal Academy of Higher Education, Manipal, Karnataka, India

I would like to commend the authors of this longitudinal study for analyzing the impact of 'peer-assisted teaching' on the English language skills of non-native English-speaking students. The study has a precise aim and methodology, and its results are reported accordingly. I find the topic exciting and relevant, as it addresses the language improvement needs of the target group.

 While the pre-post test approach used in the study provides elaborative information on the results, I suggest that the authors add information on how they validated the questionnaires. It would be helpful to know who constructed and validated them and if any pilot work was conducted.

 Moreover, I noticed that a small percentage of students disagreed with most statements about the importance of medical English skills. It would be beneficial to understand their reasons better by including open-ended response questions in future studies.

Have any limitations of the research been acknowledged?

Yes

Is the work clearly and accurately presented and does it cite the current literature?

Yes

If applicable, is the statistical analysis and its interpretation appropriate?

Yes

Are all the source data underlying the results available to ensure full reproducibility?

Yes

Is the study design appropriate and is the work technically sound?

Yes

Are the conclusions drawn adequately supported by the results?

Yes

Are sufficient details of methods and analysis provided to allow replication by others?

No

Reviewer Expertise:

Simulation, 3D printing in medical education, ethics in medical education, designing and planning learning

I confirm that I have read this submission and believe that I have an appropriate level of expertise to confirm that it is of an acceptable scientific standard, however I have significant reservations, as outlined above.
